# Glucose and Applied Voltage Accelerated *p*-Nitrophenol Reduction in Biocathode of Bioelectrochemical Systems

**DOI:** 10.3389/fmicb.2018.00580

**Published:** 2018-03-27

**Authors:** Xinyu Wang, Defeng Xing, Xiaoxue Mei, Bingfeng Liu, Nanqi Ren

**Affiliations:** State Key Laboratory of Urban Water Resources and Environment, School of Environment, Harbin Institute of Technology, Harbin, China

**Keywords:** biocathode, *p*-nitrophenol reduction, bioelectrochemical system, high-throughput sequencing, microbial community structure

## Abstract

*p*-Nitrophenol (PNP) is common in the wastewater from many chemical industries. In this study, we investigated the effect of initial concentrations of PNP and glucose and applied voltage on PNP reduction in biocathode BESs and open-circuit biocathode BESs (OC-BES). The PNP degradation efficiency of a biocathode BES with 0.5 V (Bioc-0.5) reached 99.5 ± 0.8%, which was higher than the degradation efficiency of the BES with 0 V (Bioc-0) (62.4 ± 4.5%) and the OC-BES (59.2 ± 12.5%). The PNP degradation rate constant (*k*_PNP_) of Bioc-0.5 was 0.13 ± 0.01 h^-1^, which was higher than the *k*_PNP_ of Bioc-0 (0.024 ± 0.002 h^-1^) and OC-BES (0.013 ± 0.0005 h^-1^). PNP degradation depended on the initial concentrations of glucose and PNP. A glucose concentration of 0.5 g L^-1^ was best for PNP degradation. The initial PNP increased from 50 to 130 mg L^-1^ and the *k*_PNP_ decreased from 0.093 ± 0.008 to 0.027 ± 0.001 h^-1^. High-throughput sequencing of 16S rRNA gene amplicons indicated differences in microbial community structure between BESs with different voltages and the OC-BES. The predominant populations were affiliated with *Streptococcus* (42.7%) and *Citrobacter* (54.1%) in biocathode biofilms of BESs, and *Dysgonomonas* were the predominant microorganisms in biocathode biofilms of OC-BESs. The predominant populations were different among the cathode biofilms and the suspensions. These results demonstrated that applied voltage and biocathode biofilms play important roles in PNP degradation.

## Introduction

*p*-Nitrophenol (PNP), a priority pollutant listed by the United States Environmental Protection Agency, is widely used for the synthesis of industrial products, and it is generated by the degradation of pesticides in the environment (Kowalczyk et al., 2015). In addition, PNP is known to have high toxicity, which can threaten ecosystem and human health if released directly into the environment (Chen et al., 2016). Hence, it is necessary to treat PNP-containing wastewater. The nitro group, with its high electron-withdrawing effect, and the benzene ring are resistant to oxidative degradation (Li et al., 2016); thus, an easy reduction of PNP is essential. Some physicochemical technologies have been applied to clean PNP-containing wastewater (Liu et al., 2010; An et al., 2012; Arbab Zavar et al., 2012; Yarlagadda et al., 2012; Zhang et al., 2012a; Zhou et al., 2016), which require high costs, extreme pH conditions, high power input, or a long processing time. Therefore, it is necessary to develop effective methods for PNP removal.

*p*-Nitrophenol degradation technology using the anaerobic reductive process successfully transforms PNP to *p*-aminophenol (PAP) when glucose is used as the electron donor (Sponza and Kuşçu, 2005; Kuscu and Sponza, 2007). However, the anaerobic process is usually slow and needs reductive conditions (Shen et al., 2013). In addition, a direct power supply used to enhance the degradation of difficult pollutions in anaerobic, microbial reductive processes (Zhang et al., 2012b,c; Shen et al., 2014). Bioelectrochemical systems (BESs) use electroactive microorganisms to drive electron transfer (Wang and Ren, 2013), which has been used for the removal of challenging pollutants, such as aromatic pollutants, nitrobenzene; azo dyes; nitrofurans furazolidone; cefuroxime; phenols; chloramphenicol; 2,4-dinitrochlorobenzene; and nitrate (Kong et al., 2014, 2015; Liang et al., 2014; Bajracharya et al., 2016; Cheng et al., 2016; Yun et al., 2017). BES has an advantage over conventional anaerobic treatment for recalcitrant pollutants degradation and dye decolorization (Bajracharya et al., 2016). Recently, BESs and BES-integrated conventional processes have shown excellent PNP degradation efficiency (Shen et al., 2013, 2014; Lou et al., 2015; Chen et al., 2016).

Some previous studies have shown that nitrophenols (such as PNP) were reduced to aminophenols (PAP) with less toxicity and easier mineralization (Wang et al., 2011, 2016; Shen et al., 2012; Jiang et al., 2016). The use of biocathode BESs has been reported as a low energy and sustainable method for metals remediation and nitrate remediation (Huang et al., 2015). A previous investigation reported that the cathode biofilms of biocathode BESs played an important role in PNP degradation when sodium bicarbonate was used as sole carbon source in the cathodic chamber (Wang et al., 2016). The ecological conditions affect PNP degradation and microbial community structure of the biocathode biofilm in BESs. Optimizing the operation conditions of BESs is necessary to accelerate PNP reduction velocity. Nevertheless, the functional microbial community for PNP reduction has not been fully investigated. Moreover, it is important to investigate PNP degradation efficiency and stability with different concentrations of glucose and initial PNP because PNP-containing wastewater usually has variables carbon source concentrations and PNP content.

In this study, we investigated the kinetics of PNP degradation and PAP formation in biocathode BESs. We analyzed the effects of applied voltage and the initial concentrations of glucose and PNP on PNP degradation. We also explored the biocathode microbial communities using high-throughput sequencing of 16S rRNA gene amplicons.

## Materials and Methods

### Reactor Setup

We used two-chamber BES reactors consisting of glass bottles separated by a cationic exchange membrane (Ultrex CMI7000, Membranes International, Inc., United States). The working volume of each chamber was 300 mL. Carbon brushes (5 cm in diameter and 7 cm long, fiber type: T700-12K, Toray Industries, CO., Ltd.) were used as the electrodes. Prior to use, the membrane and electrode brushes were pretreated as previously described (Wang et al., 2016). The Ag/AgCl reference electrodes [0.247 V vs. standard hydrogen electrode (SHE), model-217, Shanghai Precision Scientific Instrument Co., Ltd., China] were inserted into the cathode chambers for measuring cathode potentials and for electrochemical analysis. The anode, cathode, and reference electrodes were connected to a data acquisition system (Keithley 2700, Keithley, Co., Ltd., United States) with high-precision external resistance (10 Ω). All electric potentials reported here were already against the SHE.

### Inoculation and Operation

The reactors with biocathode were inoculated in fed-batch mode as previously described (Wang et al., 2016). PNP degradation was operated under three modes: (I) biocathode with closed circuit and 0.5 V of applied voltage (Bioc-0.5), (II) biocathode with closed circuit with 0 V of applied voltage (Bioc-0), and (III) OC-BES (as a control test). The anode anolyte culture medium contained 1.67 g L^-1^ of NaAC, trace minerals, vitamins, and 50 mM of phosphate buffer solution (PBS) (Lovley and Phillips, 1988; Lu et al., 2012). The cathode was fed with 30 mg L^-1^ PNP, trace minerals, vitamins, and 0.5 g L^-1^ glucose mixed with 50 mM PBS. We adjusted the glucose to 0.1, 0.3, 0.5, 0.8, and 1 g L^-1^ with 50 mg L^-1^ PNP to investigate the influence of different glucose concentrations. We adjusted the PNP concentration to 50, 70, 90, 110, and 130 mg L^-1^ with 0.5 g L^-1^ glucose to investigate the effects of different initial PNP concentrations. The biocathodes were replaced with new sterile carbon brushes (121°C, 30 min) to determine the impact of biocathode microbial communities on PNP degradation. The reactors and the medium were autoclaved at 121°C for 15 min. All experiments were operated in replicated cycles for consistency, and all experiments were conducted at 25 ± 2°C.

### Chemical Analyses and Calculations

The concentrations of PNP and PAP were determined, as previously reported, and the production was analyzed by a high performance liquid chromatography mass spectrometer (HPLC-MS) (Wang et al., 2016). We used a gas chromatograph (Agilent, 4890D; J&W Scientific, United States) with a flame ionization detector and an appropriate column (19095N-123HP-INNOWAX, 30 m × 0.530 mm × 1.00 μm, J&W Scientific, United States) to analyze the concentrations of volatile fatty acids (VFAs), including acetic acid, propionic acid, isobutyric acid, butyric acid, isovaleric acid, and valeric acid (Liu et al., 2016). The glucose concentration of the cathode effluent was analyzed with a glucose determination kit (RSBIO, Shanghai). We measured cell biomass of the cathode effluent with a Modified BCA Protein Assay Kit (Sangon Biotech). Before the protein tests, the effluent samples were frozen to -20°C for 24 h, then thawed and boiled for 10 min.

Current density (Am^-3^) was calculated based on the cathode volume (300 mL). PNP degradation efficiency (DE_PNP_) was calculated based on the difference between the influent and effluent PNP concentrations. The kinetics of the PNP reduction and PAP formation were assumed to follow the first-order reaction models *C* = *C*_0_*e^-kt^* and *C* = *C*_0_(1-*e^-kt^*), respectively (*C* represents the PNP or PAP concentration (mg L*^-^*^1^) at time (h) and *C*_0_ is the initial PNP concentration or maximum PAP concentration); the rate constant *k* (h*^-^*^1^) of PNP and PAP was calculated by Origin 8.0 software. The half-life time (*t*_1/2_) of PNP was calculated using the equation *t*_1/2_ = 0.693/*k*.

### Electrochemical Analysis

We conducted cyclic voltammetry (CV) on the cathode using an electrochemical workstation (WMPG1000K8 multichannel potentiostat, WonATech, Co., Ltd., South Korea); the anode was the counter electrode, and Ag/AgCl was the reference electrode (+0.197 V vs. SHE). We measured CV for 30 mg L*^-^*^1^ of PNP and 0.5 g L*^-^*^1^ of glucose at a scan rate of 5 mV/s. All CV tests were operated at 25°C with a scan range from -1.0 to +1.0 V. We used electrochemical impedance spectroscopy (EIS) with the same instrument as the CV tests with a frequency range from 100 KHz to 10 mHz using a 10 mV sine wave.

### Microbial Community Analysis

Samples of cathodic biofilms (Bioc-0.5-C, Bioc-0-C, and OC-BES-C) and suspended growth cultures (Bioc-0.5-S, Bioc-0-S, and OC-BES-S) were removed aseptically from the corresponding reactors. DNA was extracted using the PowerSoil DNA Isolation Kit (MO BIO, Carlsbad, CA, United States). DNA samples were stored at -20°C before analysis. Polymerase chain reaction (PCR) amplifications of bacteria were sequenced using the universal primers 8F (5′-AGAGTTTGATCCTGGCTCAG-3′) and 533R (5′-TTACCGCGGCTGCTGGCAC-3′) for the 16S rRNA gene V1–V3 region (length of approximately 455 bp). We conducted 454 GS-FLX pyrosequencing using the method described previously (Wang et al., 2016). The abundance of a given phylogenetic group was defined by the proportion of the number of sequences affiliated to that group to the total number of sequences obtained, and we conducted 454 pyrosequencing data analyses using the methods detailed in our previous study (Wang et al., 2016).

## Results

### PNP Degradation in Biocathode BESs

**FIGURE 1:**
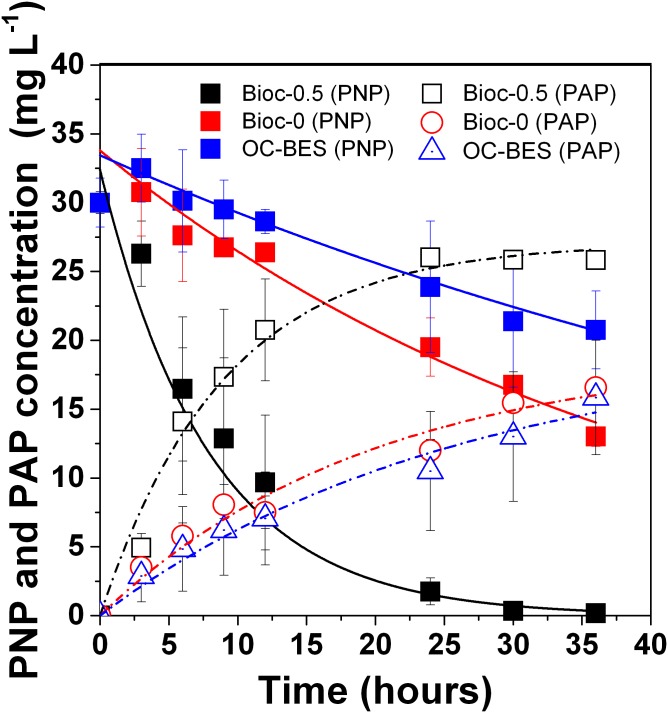
*p*-Nitrophenol (PNP) degradation and PAP formation in biocathode BES under different modes, with initial PNP 30 mg L^-1^ and glucose 0.5 g L^-1^. Error bars represent standard deviation (SD) based on three tests. The Bioc-0.5 was the biocathode BES with 0.5 V voltage, Bioc-0 was t biocathode BES with 0 V voltage, OC-BES was BES with open circuit.

The PNP degradation rate (*k*_PNP_) and PAP formation rate (*k*_PAP_) were fitted with first-order kinetics (all *R*^2^> 0.96) (Supplementary Table S1). With the applied voltage, the PNP degradation rate (*k*_PNP_) of Bioc-0.5 was 0.13 ± 0.01 h^-1^ (*R*^2^ = 0.996), which was five times greater than that of Bioc-0 (0.024 ± 0.002 h^-1^, *R*^2^ = 0.977), and almost 10 times greater than that of OC-BES (0.013 ± 0.005 h^-1^, *R*^2^ = 0.991). The PAP formation rate (*k*_PAP_) followed the same trend and the values were 0.11 ± 0.01 (*R*^2^ = 0.983), 0.051 ± 0.012 (*R*^2^ = 0.967), and 0.038 ± 0.011 (*R*^2^ = 0.972) for Bioc-0.5, Bioc-0, and OC-BES, respectively. The PNP degradation efficiency (DE_PNP_) of Bioc-0.5 at 36 h was 99.5 ± 0.8%, which was significantly higher than the DE_PNP_ of Bioc-0 (60.9 ± 0.05%) and OC-BES (37.7 ± 5.2%) (**Figure 1** and Supplementary Table S1). These results indicated that applied voltage significantly enhanced PNP degradation.

**FIGURE 2:**
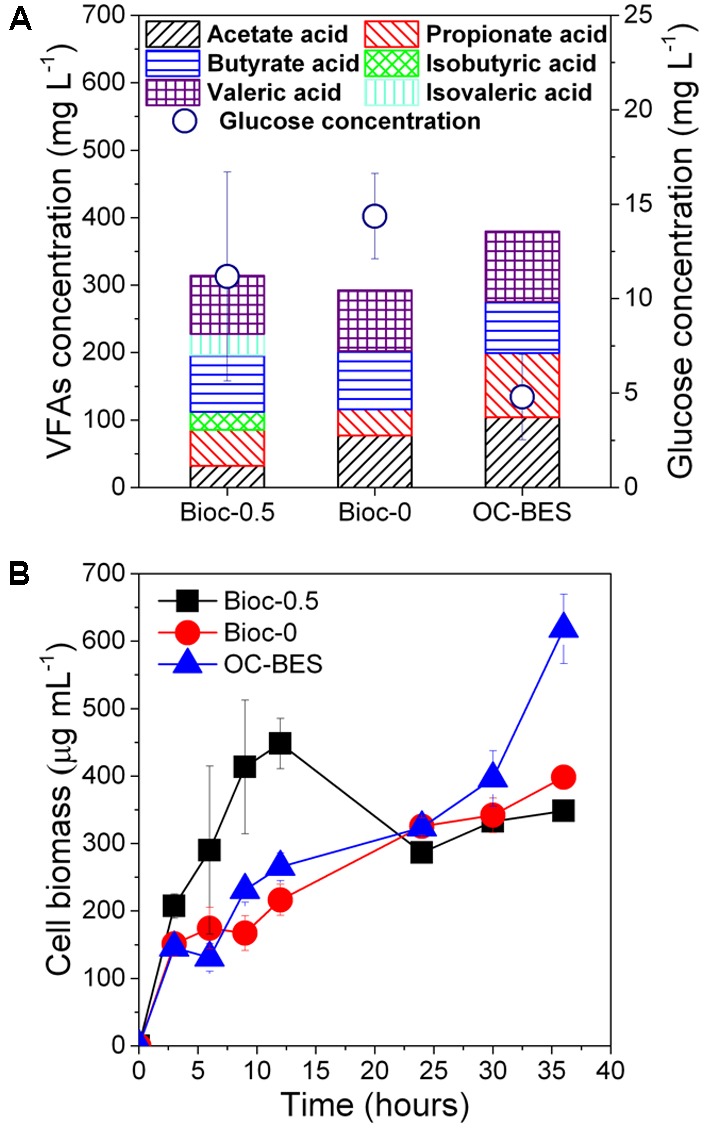
The concentrations of glucose and volatile fatty acids (VFAs) **(A)** of 6 h and the biomass of the cathode effluent **(B)**. Error bars represent standard deviation (SD) based on three tests. The Bioc-0.5 was the biocathode BES with 0.5 V voltage, Bioc-0 was the biocathode BES with 0 V voltage, OC-BES was BES with open circuit.

In all experiments, glucose was consumed quickly and the concentrations were less than 11.4 ± 2.2 mg L^-1^ within 6 h (**Figure 2A**). The concentration of VFAs of Bioc-0.5 was lower than the VFA concentration of OC-BES and higher than the VFA concentration of Bioc-0. Abundant cell biomass was present in the suspension of cathodic chamber (**Figure 2B**), and the cell biomass of the effluent was different among three modes. The cell biomass of Bioc-0.5 increased in the first 12 h, then decreased from 12 to 24 h, and the cell biomass was stable at the final stage (from 24 to 36 h). The cell biomass of Bioc-0 and OC-BES increased gradually, and the cell biomass was greatest in the OC-BES.

### Electrochemical Properties of Biocathode BESs for PNP Removal

**FIGURE 3:**
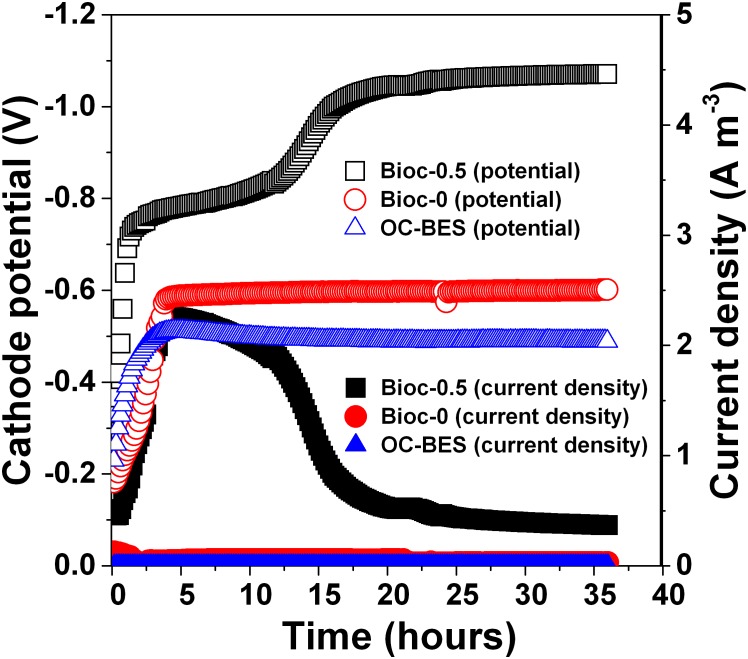
The dynamic changes of the cathode potential and current density during PNP degradation under different operational modes. The Bioc-0.5 was the biocathode BES with 0.5 V voltage, Bioc-0 was the biocathode BES with 0 V voltage, OC-BES was BES with open circuit.

The cathode potential decreased and stabilized after 20 h at approximately -1.0 V for Bioc-0.5, which was lower than the cathode potential of Bioc-0 (-580 mV) and OC-BES (-493 mV) (**Figure 3**). The current density of Bioc-0.5 reached 2.49–2.26 Am^-3^, which was much higher than the current density of Bioc-0 (approximately 0.057 Am^-3^). After reaching the maximum, the current density of Bioc-0.5 decreased and stabilized at approximately 0.5 Am^-3^ after 20 h; the current density of Bioc-0 was approximately 0.028 Am^-3^. These results showed that applied voltage can significantly increase the absolute cathodic potential and the current density.

No redox peak was observed from the cathode of Bioc-0.5 and Bioc-0, but the polarization currents changed significantly (Supplementary Figure S1A). Compared to the CV curve of Bioc-0, the cathodic current of Bioc-0.5 was enhanced, and the onset potential had a positive shift with an application of 0.5 V. The EIS analysis indicated that the internal resistance of Bioc-0.5 was 391 Ω, which was 23% less than the internal resistance of Bioc-0 (508 Ω) (Supplementary Figure S1B).

### Effect of an Exogenous Carbon Source on PNP Reduction

**FIGURE 4:**
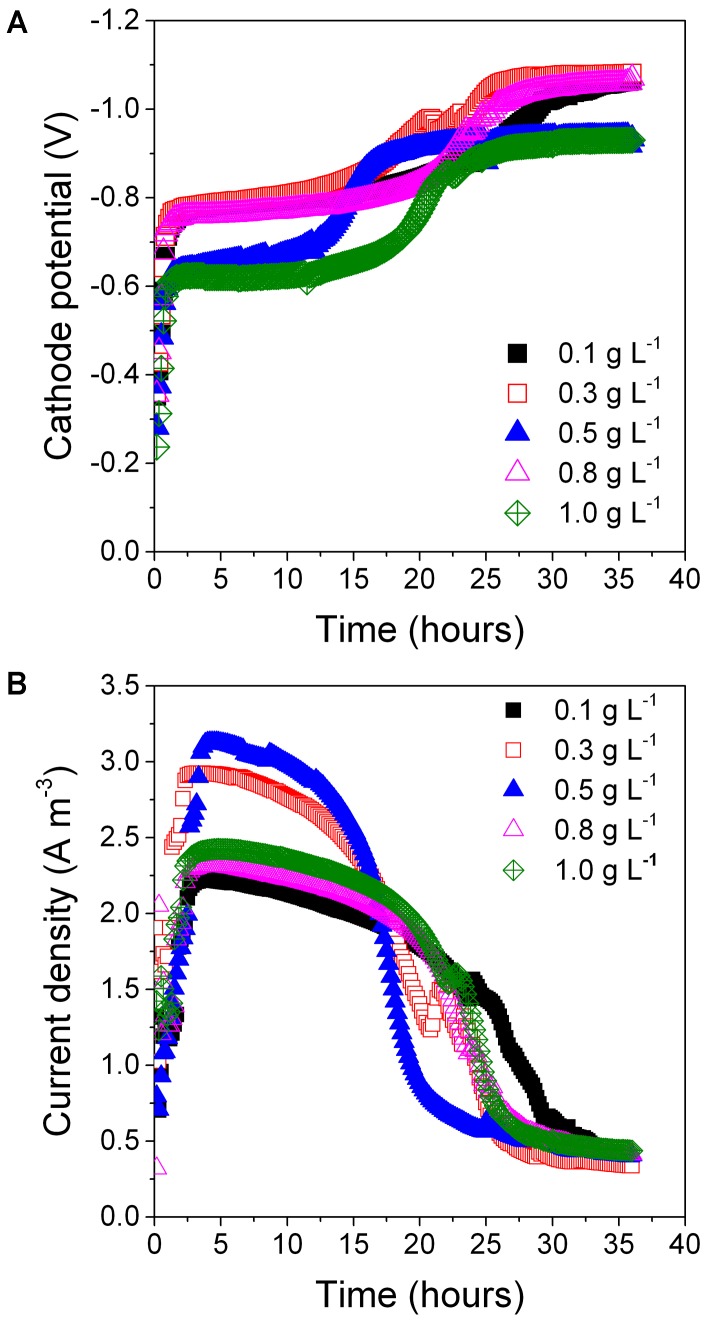
The cathode potential **(A)** and current density **(B)** at different glucose concentration on PNP reduction.

Different initial concentrations of the exogenous carbon source substantially influenced PNP reduction. With the addition of 0.5 g L^-1^ of glucose *k*_PNP_, *k*_PAP_, and DE_PNP_ were 0.093 ± 0.008 h^-1^ (*R*^2^ = 0.982), 0.086 ± 0.009 h^-1^ (*R*^2^ = 0.987), and 99.8 ± 0.35%, respectively (**Table 1**). A higher glucose concentration (>0.5 g L^-1^) resulted in PNP degradation (0.070 ± 0.009–0.084 ± 0.008) decreased and a lower glucose concentration (<0.5 g L^-1^) resulted in decreased PAP formation (0.0391 ± 0.00945–0.0509 ± 0.0111). All samples had similar cathode potentials (all approximately -1.0 V) (**Figure 4A**). Maximum peak current density was at 0.5 g L^-1^ glucose (3.1 Am^-3^) (**Figure 4B**). The peak current density of other glucose concentrations decreased to <2.9 Am^-3^. These results showed that 0.5 g L^-1^ was an optimum glucose concentration for PNP degradation.

**Table 1:** The *k*_PNP_, *R^2^*_PNP_, *t*_1/2PNP_, DE_PNP_, *k*_PAP_, *R^2^*_PAP_ of different glucose concentration on PNP degradation.

Glucose (g L^-1^)	*k*_PNP_ (h^-1^)	*R*^2^_PNP_	*t*_1/2PNP_	DE_PNP_ (%)	*k*_PAP_ (h^-1^)	*R*^2^_PAP_
0.1	0.0699 ± 0.00906	0.958	9.92	97.7 ± 0.36	0.0391 ± 0.00945	0.980
0.3	0.0843 ± 0.00856	0.976	8.22	98.8 ± 0.34	0.0647 ± 0.00851	0.986
0.5	0.0933 ± 0.0082	0.982	7.42	99.8 ± 0.35	0.0865 ± 0.00914	0.987
0.8	0.0787 ± 0.007	0.980	8.802	94.8 ± 0.31	0.0573 ± 0.00721	0.989
1	0.0719 ± 0.00875	0.962	9.64	96.9 ± 0.34	0.0509 ± 0.0111	0.975


Current and cathode potential increased when the biofilms were eliminated from the cathode (Supplementary Figure S2). The PNP degradation rate (*k*_PNP_) decreased from 0.098 ± 0.011 to 0.022 ± 0.001 (Supplementary Figure S3). PNP removal efficiency decreased from 98.4 ± 1.1 to 40.1 ± 2.8%, and the PAP concentration of the effluent decreased from 26.5 ± 0.4 to 4.5 ± 1.0 mg L^-1^ at 24 h with no biofilm (Supplementary Figure S4). These results showed that the electrode biofilm contributed to PNP removal.

### Effect of Initial PNP Concentration on PNP Reduction

**FIGURE 5:**
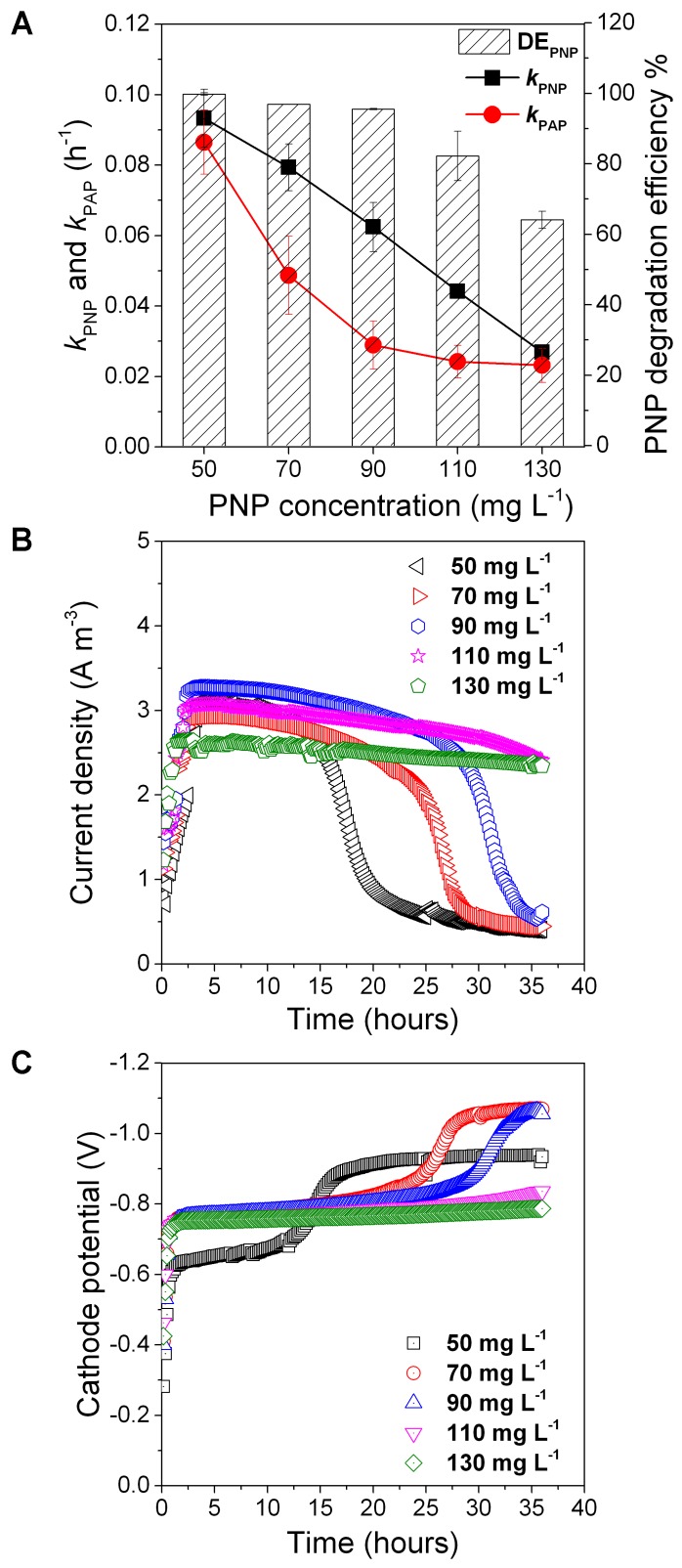
The PNP degradation rate, PAP formation rate and PNP degradation efficiency **(A)** and cathode potential **(B)** and current density **(C)** at different initial PNP concentration. Error bars represent standard deviation (SD) based on three tests.

Five initial concentrations of PNP were used to assess PNP reduction with 0.5 g L^-1^ glucose (**Figure 5A**). We found that *k*_PNP_ decreased from 0.093 ± 0.008 to 0.027 ± 0.0001, *k*_PAP_ decreased from 0.086 ± 0.00914 to 0.0231 ± 0.005, and DE_PNP_ decreased from 99.8 ± 0.35 to 64.1 ± 2.4% when the initial concentration of PNP increased from 50 to 130 mg L^-1^ (**Figure 5A**). As the PNP concentration increased from 50 to 130 mg L^-1^, the cathode potential and the current density increased and then decreased (**Figures 5B,C**). A lower initial concentration of 30 mg/L showed a higher *k*_PNP_ (0.13 ± 0.01 h^-1^) (**Figure 1**). These results showed that the initial PNP concentration influenced the rate of PNP reduction and its efficiency.

### Microbial Community Structure in Biocathode BESs

Total operational taxonomic units (OTUs) of 440–545 were obtained from the clean reads of 7833–11121 (Supplementary Table S2). Microbial communities of Bioc-0.5-S and Bioc-0.5-C had higher species richness, and Bioc-0-C and Bioc-0-S had higher species diversity. A principal component analysis (PCA) (Supplementary Figure S5) indicated that Bioc-0.5-C and Bioc-0.5-S were clustered together. In Bioc-0 and OC-BES, the community compositions of the suspension and biocathode biofilm were dissimilar.

**FIGURE 6:**
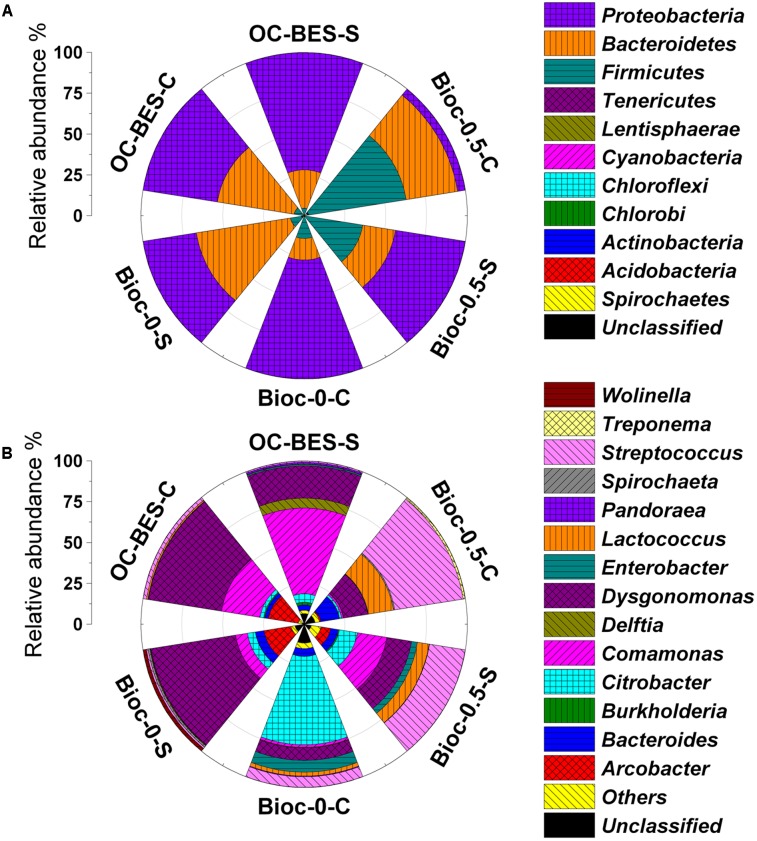
Relative abundance of predominant phylum **(A)** and genera **(B)** in the cathode suspension and biofilm of BES. The “others” was the phyla and genera less than 1% of the total summarized. The Bioc-0.5 was the biocathode BES with 0.5 V voltage, Bioc-0 was the biocathode BES with 0 V voltage, OC-BES was BES with open circuit. C represents cathode and S represents suspension.

The microbial communities of six samples were dominated by the phyla *Firmicutes*, *Bacteroidetes*, and *Proteobacteria* (**Figure 6A**). The predominant phyla of Bioc-0.5-C was *Firmicutes* (60.4%) and *Bacteroidetes* (30.9%); *Proteobacteria* (43.4%) and *Firmicutes* (35.7%) were the predominant phyla in Bioc-0.5-S. By contrast, the predominant phyla were *Proteobacteria* (72.8%) for Bioc-0-C and *Bacteroidetes* (58.3%) and *Proteobacteria* (33.1%) for Bioc-0-S. In OC-BES, the main phyla were *Bacteroidetes* (47.6%) and *Proteobacteria* (46.0%) for OC-BES-C and *Proteobacteria* (71.8%) and *Bacteroidetes* (23.6%) for OC-BES-S. At the family level, *Streptococcaceae* was predominant in Bioc-0.5-C (58.4%) and Bioc-0.5-S (29.6%); *Porphyromonadaceae* was predominant in OC-BES-C (45.2%) and Bioc-0-S (52.5%); *Enterobacteriaceae* was dominant in Bioc-0-C (65.9%) (Supplementary Figure S6).

Most populations of bacteria in Bioc-0.5-C were affiliated with *Streptococcus* (42.7%), *Lactococcus* (15.6%), and *Bacteroides* (11.4%). *Streptococcus* (22.2%), *Comamonas* (18.1%), and *Citrobacter* (11.2%) were the predominant populations in Bioc-0.5-S (**Figure 6B**). Bioc-0-C was mostly *Citrobacter* (54.1%) bacteria, and Bioc-0-S was mostly *Dysgonomonas* (52.3%) bacteria. By contrast, the relative abundance of *Dysgonomonas* and *Comamonas* were 45.2 and 24.1%, respectively, in OC-BES-C and 19.7 and 52.4%, respectively, in OC-BES-S. These results indicated that power supply greatly affected the community composition of the electrode biofilms and suspensions.

## Discussion

This study proved BESs substantially enhanced PNP degradation. Compared to a previous study, *k*_PNP_ (0.093 ± 0.008 h^-1^) of BESs fed with glucose was approximately three times greater than the *k*_PNP_ of BESs fed with sodium bicarbonate (0.02978 ± 0.00339 h^-1^) (Wang et al., 2016). More importantly, electrodes of the biocathode were not the sole electron donor to PNP, glucose was also an electron donor for PNP degradation. In the anodic chamber, H^+^ and electron were generated by NaAC and were transferred to the cathodic chamber as the electron for PNP reduction. The glucose in the cathodic chamber could also generate *e*^-^, and PNP was reduced to PAP in the cathodic chamber.

In the cathodic chamber, glucose was first transformed by the bacteria to relatively lower molecular VFAs through a fermentation process (**Figure 2A**). The VFAs were then used as the electron donors for PNP degradation after the glucose was consumed (6 h), which was called a syntrophic interaction in a previous study (Zeng et al., 2015). Syntrophic relationships between fermentative and PNP-reducing bacteria were essential in the biocathode when the glucose was fully consumed within 6 h (for an initial glucose concentration of 0.5 g L^-1^). PNP degradation in the OC-BES further indicated that partial PNP reduction was by glucose and not by the cathode.

A previous study indicated that PNP can inhibit PNP biodegradation because it is toxic to bacteria (Carrera et al., 2011). A high concentration of PNP (over 90 mg/L) could depress bacteria biofilms and the performance of BES. In our study, PNP reduction occurred from 30 to 130 mg L^-1^, further demonstrating the advantage of PNP tolerance in biocathode BES for PNP degradation. The biofilm removed from the biocathode of BESs, the PNP degradation and the PAP formation decreased (Supplementary Figures S3, S4), implied that biofilms on the cathode and the applied voltage influenced PNP degradation. The current of the BES with an abiotic cathode was lower than the BES with a biotic cathode, indicating that the biocathode can supply electrons for PNP reduction.

*p*-Nitrophenol-degrading bacteria have been isolated from enrichment cultures to reveal whether the same microorganisms are responsible for PNP degradation in the natural environment (Kowalczyk et al., 2015). Most PNP-degrading bacteria can use PNP as their sole source of carbon, nitrogen, and energy. The detected genera in this study were mainly related to exoelectrogens. *Pseudomonas* (Kulkarni and Chaudhari, 2006; Kowalczyk et al., 2015), *Arthrobacter* (Sahoo et al., 2011; Wang et al., 2015), *Flavobacterium* (Sahoo et al., 2011), *Achromobacter* (Wan et al., 2007), *Sphingomonas* (Leung et al., 1997), *Burkholderia* (Pandey et al., 2012), and *Stenotrophomonas* (Liu et al., 2007) are PNP-degrading bacteria whose relative abundance was lower in Bioc-0.5-C (0.15–2.3%), suggesting that a large number of unknown PNP-degrading bacteria may be enriched in BESs.

The relative abundance of *Streptococcus* was highest in the biocathode biofilms of Bioc-0.5-C. *Streptococcus*, a biofilm-forming pathogen, has been studied (Loo et al., 2000; Moscoso et al., 2006), but its capability of PNP degradation is not known. *Dysgonomonas* was present in the suspension and the OC-BES cathode, and it was the predominant genus detected in the BESs (Watanabe et al., 2011; Kodama et al., 2012). The electroactive *Comamonas*, enriched in the biofilms and suspension of OC-BESs, can use phenol, 4-nitrobenzoate, 4-chlorophenol, and nitrobenzene (Groenewegen and Debont, 1992; Hollender et al., 1997; Arai et al., 2000; Wu et al., 2006). The PNP degradation of *Streptococcus, Dysgonomonas*, and *Comamonas* should be investigated in the future. Revealing functional genes related with PNP degradation and syntrophic interaction between different populations using metagenomic technology is still important to understand PNP degradation in future study on BES.

## Conclusion

*p*-Nitrophenol degradation was enhanced in the biocathode BESs with glucose and an applied voltage of 0.5 V. PNP degradation efficiency of the BES was much higher than that of OC-BES. The initial concentrations of glucose and PNP influenced PNP degradation and PAP formation. The microbial communities of the biocathode biofilm and suspension were different in OC-BES and BES with different voltages, implying that the differences in microbial communities and BES resulted in different PNP degradation. These results demonstrated that voltage and biocathode biofilms contribute to PNP degradation.

## Author Contributions

DX and NR designed the experiments. XW performed the experiments. XW, DX, XM, and BL contributed the data analysis and wrote sections of the manuscript. All authors contributed to manuscript revision and approved the submitted version.

## Conflict of Interest Statement

The authors declare that the research was conducted in the absence of any commercial or financial relationships that could be construed as a potential conflict of interest.

## References

[B1] An F. Du R. Wang X. Wan M. Dai X. Gao J. (2012). Adsorption of phenolic compounds from aqueous solution using salicylic acid type adsorbent. *J. Hazard. Mater.* 201–202 74–81. 10.1016/j.jhazmat.2011.11.037 22169143

[B2] Arai H. Ohishi T. Chang M. Y. Kudo T. (2000). Arrangement and regulation of the genes for meta-pathway enzymes required for degradation of phenol in *Comamonas testosteroni* TA441. *Microbiology* 146 1707–1715. 10.1099/00221287-146-7-1707 10878134

[B3] Arbab Zavar M. H. Heydari S. Rounaghi G. H. Eshghi H. Azizi-Toupkanloo H. (2012). Electrochemical behavior of *para*-nitroaniline at a new synthetic crown ether-silver nanoparticle modified carbon paste electrode. *Anal. Methods* 4 953–958. 10.1039/c2ay05892h

[B4] Bajracharya S. Sharma M. Mohanakrishna G. Dominguez Benneton X. Strik D. P. B. T. B. Sarma P. M. (2016). An overview on emerging bioelectrochemical systems (BESs): technology for sustainable electricity, waste remediation, resource recovery, chemical production and beyond. *Renew. Energy* 98 153–170. 10.1016/j.renene.2016.03.002

[B5] Carrera J. Martin-Hernandez M. Suarez-Ojeda M. E. Perez J. (2011). Modelling the pH dependence of the kinetics of aerobic *p*-nitrophenol biodegradation. *J. Hazard. Mater.* 186 1947–1953. 10.1016/j.jhazmat.2010.12.096 21247692

[B6] Chen X. Murugananthan M. Zhang Y. (2016). Degradation of p-Nitrophenol by thermally activated persulfate in soil system. *Chem. Eng. J.* 283 1357–1365. 10.1016/j.cej.2015.08.107

[B7] Cheng Z. Hu X. Sun Z. (2016). Microbial community distribution and dominant bacterial species analysis in the bio-electrochemical system treating low concentration cefuroxime. *Chem. Eng. J.* 303 137–144. 10.1016/j.cej.2016.05.131

[B8] Groenewegen P. E. J. Debont J. A. M. (1992). Degradation of 4-nitrobenzoate via 4-hydroxylaminobenzoate and 3,4-dihydroxybenzoate in *Comamonas acidovorans* NBA-10. *Arch. Microbiol.* 158 381–386. 10.1007/bf00245369 1527502

[B9] Hollender J. Hopp J. Dott W. (1997). Degradation of 4-chlorophenol via the *meta* cleavage pathway by *Comamonas testosteroni* JH5. *Appl. Environ. Microbiol.* 63 4567–4572. 16535738 10.1128/aem.63.11.4567-4572.1997 PMC1389294

[B10] Huang L. Wang Q. Jiang L. Zhou P. Quan X. Logan B. E. (2015). Adaptively evolving bacterial communities for complete and selective reduction of Cr(VI), Cu(II), and Cd(II) in biocathode bioelectrochemical systems. *Environ. Sci. Technol.* 49 9914–9924. 10.1021/acs.est.5b00191 26175284

[B11] Jiang X. Shen J. Lou S. Mu Y. Wang N. Han W. (2016). Comprehensive comparison of bacterial communities in a membrane-free bioelectrochemical system for removing different mononitrophenols from wastewater. *Bioresour. Technol.* 216 645–652. 10.1016/j.biortech.2016.06.005 27289055

[B12] Kodama Y. Shimoyama T. Watanabe K. (2012). *Dysgonomonas oryzarvi* sp. nov., isolated from a microbial fuel cell. *Int. J. Syst. Evol. Microbiol.* 62 3055–3059. 10.1099/ijs.0.039040-0 22307505

[B13] Kong D. Liang B. Yun H. Ma J. Li Z. Wang A. (2015). Electrochemical degradation of nitrofurans furazolidone by cathode: characterization, pathway and antibacterial activity analysis. *Chem. Eng. J.* 262 1244–1251. 10.1016/j.cej.2014.10.094

[B14] Kong F. Wang A. Cheng H. Liang B. (2014). Accelerated decolorization of azo dye Congo red in a combined bioanode-biocathode bioelectrochemical system with modified electrodes deployment. *Bioresour. Technol.* 151 332–339. 10.1016/j.biortech.2013.10.027 24262842

[B15] Kowalczyk A. Eyice O. Schafer H. Price O. R. Finnegan C. J. van Egmond R. A. (2015). Characterization of *para*-Nitrophenol-degrading bacterial communities in river water by using functional markers and stable isotope probing. *Appl. Environ. Microbiol.* 81 6890–6900. 10.1128/AEM.01794-15 26209677 PMC4561691

[B16] Kulkarni M. Chaudhari A. (2006). Biodegradation of *p*-nitrophenol by *P. putida*. *Bioresour. Technol.* 97 982–988. 10.1016/j.biortech.2005.04.036 16009549

[B17] Kuscu O. S. Sponza D. T. (2007). Performance of *p*-nitrophenol (*p*-NP) fed sequential anaerobic migrating blanket reactor (AMBR)/aerobic completely stirred tank reactor (CSTR) system under increasing organic loading conditions. *Enzyme Microb. Technol.* 40 1026–1034. 10.1016/j.enzmictec.2006.08.001

[B18] Leung K. T. Tresse O. Errampalli D. Lee H. Trevors J. T. (1997). Mineralization of *p*-nitrophenol by pentachlorophenol-degrading *Sphingomonas* spp. *FEMS Microbiol. Lett.* 155 107–114. 10.1111/j.1574-6968.1997.tb12693.x

[B19] Li L. Liu Q. Wang Y. X. Zhao H. Q. He C. S. Yang H. Y. (2016). Facilitated biological reduction of nitroaromatic compounds by reduced graphene oxide and the role of its surface characteristics. *Sci. Rep.* 6:30082. 10.1038/srep30082 27439321 PMC4954959

[B20] Liang B. Cheng H. Van Nostrand J. D. Ma J. Yu H. Kong D. (2014). Microbial community structure and function of Nitrobenzene reduction biocathode in response to carbon source switchover. *Water Res.* 54 137–148. 10.1016/j.watres.2014.01.052 24565804

[B21] Liu Q. Ren Z. J. Huang C. Liu B. Ren N. Xing D. (2016). Multiple syntrophic interactions drive biohythane production from waste sludge in microbial electrolysis cells. *Biotechnol. Biofuels* 9:162. 10.1186/s13068-016-0579-x 27489567 PMC4971668

[B22] Liu Y. Wang D. Sun B. Zhu X. (2010). Aqueous 4-nitrophenol decomposition and hydrogen peroxide formation induced by contact glow discharge electrolysis. *J. Hazard. Mater.* 181 1010–1015. 10.1016/j.jhazmat.2010.05.115 20576351

[B23] Liu Z. Yang C. Qiao C. (2007). Biodegradation of *p*-nitrophenol and 4-chlorophenol by *Stenotrophomonas* sp. *FEMS Microbiol. Lett.* 277 150–156. 10.1111/j.1574-6968.2007.00940.x 18031334

[B24] Loo C. Y. Corliss D. A. Ganeshkumar N. (2000). *Streptococcus gordonii* biofilm formation: identification of genes that code for biofilm phenotypes. *J. Bacteriol.* 182 1374–1382. 10.1128/jb.182.5.1374-1382.2000 10671461 PMC94426

[B25] Lou S. Jiang X. B. Chen D. Shen J. Y. Han W. Q. Sun X. Y. (2015). Enhanced *p*-nitrophenol removal in a membrane-free bio-contact coupled bioelectrochemical system. *RSC Adv.* 5 27052–27059. 10.1039/c4ra17218c

[B26] Lovley D. R. Phillips E. J. P. (1988). Novel mode of microbial energy metabolism: organic carbon oxidation coupled to dissimilatory reduction of iron or manganese. *Appl. Environ. Microbiol.* 54 1472–1480. 16347658 10.1128/aem.54.6.1472-1480.1988 PMC202682

[B27] Lu L. Xing D. Ren N. Logan B. E. (2012). Syntrophic interactions drive the hydrogen production from glucose at low temperature in microbial electrolysis cells. *Bioresour. Technol.* 124 68–76. 10.1016/j.biortech.2012.08.040 22989636

[B28] Moscoso M. Garcia E. Lopez R. (2006). Biofilm formation by *Streptococcus pneumoniae*: role of choline, extracellular DNA, and capsular polysaccharide in microbial accretion. *J. Bacteriol.* 188 7785–7795. 10.1128/JB.00673-06 16936041 PMC1636320

[B29] Pandey J. Sharma N. K. Khan F. Ghosh A. Oakeshott J. G. Jain R. K. (2012). Chemotaxis of *Burkholderia* sp. strain SJ98 towards chloronitroaromatic compounds that it can metabolise. *BMC Microbiol.* 12:19. 10.1186/1471-2180-12-19 22292983 PMC3293717

[B30] Sahoo N. K. Pakshirajan K. Ghosh P. K. (2011). Biodegradation of *p*-nitrophenol using *Arthrobacter chlorophenolicus A6* in a novel upflow packed bed reactor. *J. Hazard. Mater.* 190 729–737. 10.1016/j.jhazmat.2011.03.106 21501928

[B31] Shen J. Feng C. Zhang Y. Jia F. Sun X. Li J. (2012). Bioelectrochemical system for recalcitrant *p*-nitrophenol removal. *J. Hazard. Mater.* 209–210 516–519. 10.1016/j.jhazmat.2011.12.065 22277341

[B32] Shen J. Xu X. Jiang X. Hua C. Zhang L. Sun X. (2014). Coupling of a bioelectrochemical system for *p*-nitrophenol removal in an upflow anaerobic sludge blanket reactor. *Water Res.* 67 11–18. 10.1016/j.watres.2014.09.003 25259679

[B33] Shen J. Zhang Y. Xu X. Hua C. Sun X. Li J. (2013). Role of molecular structure on bioelectrochemical reduction of mononitrophenols from wastewater. *Water Res.* 47 5511–5519. 10.1016/j.watres.2013.06.025 23863387

[B34] Sponza D. T. Kuşçu Ö. S. (2005). *p*-Nitrophenol removal in a sequential anaerobic migrating blanket reactor (AMBR)/aerobic completely stirred tank reactor (CSTR) system. *Process Biochem.* 40 1679–1691. 10.1016/j.procbio.2004.06.063 21295402

[B35] Wan N. Gu J. D. Yan Y. (2007). Degradation of *p*-nitrophenol by *Achromobacter xylosoxidans* Ns isolated from wetland sediment. *Int. Biodeterior. Biodegradation* 59 90–96. 10.1016/j.ibiod.2006.07.012

[B36] Wang A. J. Cheng H. Y. Liang B. Ren N. Q. Cui D. Lin N. (2011). Efficient reduction of nitrobenzene to aniline with a biocatalyzed cathode. *Environ. Sci. Technol.* 45 10186–10193. 10.1021/es202356w 21985580

[B37] Wang H. Ren Z. J. (2013). A comprehensive review of microbial electrochemical systems as a platform technology. *Biotechnol. Adv.* 31 1796–1807. 10.1016/j.biotechadv.2013.10.001 24113213

[B38] Wang J. Ren L. Jia Y. Ruth N. Shi Y. Qiao C. (2015). Degradation characteristics and metabolic pathway of 4-nitrophenol by a halotolerant bacterium *Arthrobacter* sp. CN2. *Toxicol. Environ. Chem.* 98 226–240. 10.1080/02772248.2015.1115507

[B39] Wang X. Xing D. Ren N. (2016). *p*-Nitrophenol degradation and microbial community structure in a biocathode bioelectrochemical system. *RSC Adv.* 6 89821–89826. 10.1039/C6RA17446A

[B40] Watanabe K. Miyahara M. Shimoyama T. Hashimoto K. (2011). Population dynamics and current-generation mechanisms in cassette-electrode microbial fuel cells. *Appl. Microbiol. Biotechnol.* 92 1307–1314. 10.1007/s00253-011-3598-3 21983705

[B41] Wu J. F. Jiang C. Y. Wang B. J. Ma Y. F. Liu Z. P. Liu S. J. (2006). Novel partial reductive pathway for 4-chloronitrobenzene and nitrobenzene degradation in *Comamonas* sp. strain CNB-1. *Appl. Environ. Microbiol.* 72 1759–1765. 10.1128/AEM.72.3.1759-1765.2006 16517619 PMC1393224

[B42] Yarlagadda V. N. Kadali R. Sharma N. Sekar R. Vayalam Purath V. (2012). Rapid establishment of *p*-nitrophenol biodegradation in acetate-fed aerobic granular sludge. *Appl. Biochem. Biotechnol.* 166 1225–1235. 10.1007/s12010-011-9509-3 22205323

[B43] Yun H. Liang B. Kong D. Y. Cheng H. Y. Li Z. L. Gu Y. B. (2017). Polarity inversion of bioanode for biocathodic reduction of aromatic pollutants. *J. Hazard. Mater.* 331 280–288. 10.1016/j.jhazmat.2017.02.054 28273578

[B44] Zeng X. Borole A. P. Pavlostathis S. G. (2015). Biotransformation of furanic and phenolic compounds with hydrogen gas production in a microbial electrolysis cell. *Environ. Sci. Technol.* 49 13667–13675. 10.1021/acs.est.5b02313 26503792

[B45] Zhang A. Wang N. Zhou J. Jiang P. Liu G. (2012a). Heterogeneous Fenton-like catalytic removal of *p*-nitrophenol in water using acid-activated fly ash. *J. Hazard. Mater.* 201–202 68–73. 10.1016/j.jhazmat.2011.11.033 22169244

[B46] Zhang J. Zhang Y. Quan X. (2012b). Electricity assisted anaerobic treatment of salinity wastewater and its effects on microbial communities. *Water Res.* 46 3535–3543. 10.1016/j.watres.2012.03.059 22516174

[B47] Zhang J. Zhang Y. Quan X. Li Y. Chen S. Zhao H. (2012c). An anaerobic reactor packed with a pair of Fe-graphite plate electrodes for bioaugmentation of azo dye wastewater treatment. *Biochem. Eng. J.* 63 31–37. 10.1016/j.bej.2012.01.008

[B48] Zhou H. Hu L. Wan J. Yang R. Yu X. Li H. (2016). Microwave-enhanced catalytic degradation of *p*-nitrophenol in soil using MgFe_2_O_4_. *Chem. Eng. J.* 284 54–60. 10.1016/j.cej.2015.08.103

