# Supplementary material for: Glucose and Applied Voltage Accelerated p-Nitrophenol Reduction in Biocathode of Bioelectrochemical Systems
Source: Front Microbiol. 2018 Mar 27;9:580. doi: 10.3389/fmicb.2018.00580 (PMC5881249; doi:10.3389/fmicb.2018.00580)
Supplement: Supplementary file 1 [file Data_Sheet_1.pdf]

frontiers

Supplementary Material

Glucose and Applied Voltage Accelerated p-Nitrophenol Reduction in Biocathode of Bioelectrochemical Systems

Xinyu Wang , Defeng Xing*, Xiaoxue Mei, Bingfeng Liu, Nanqi Ren*

*** Correspondence:** Prof. Defeng Xing: dxing@hit.edu.cn, Prof. Nanqi Ren: rnq@hit.edu.cn

1 SUPPLEMENTARY FIGURES AND TABLES

1.1 Supplementary Figures

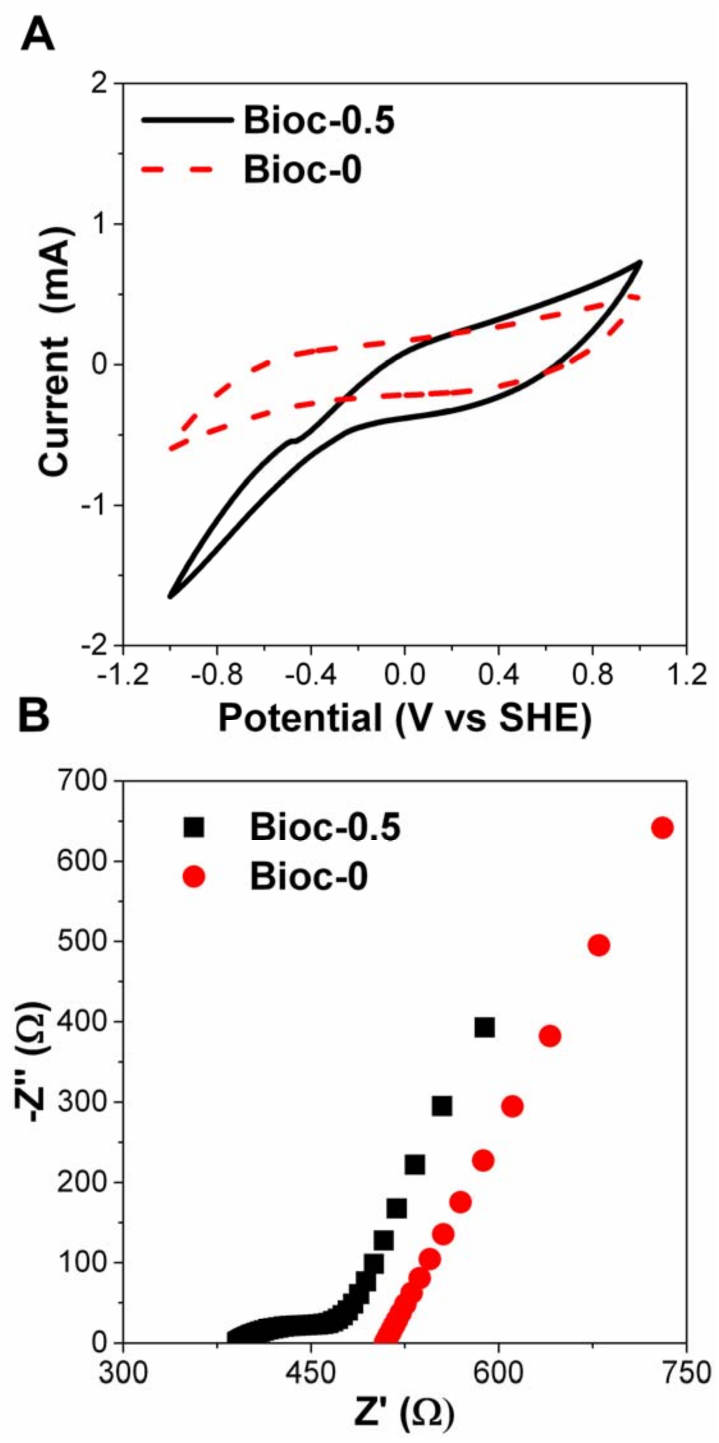

Figure S1: Cyclic voltammograms (A) in fresh medium with sacn rates of 5 mV per second and cathode EIS (B). The Bioc-0.5 was the biocathode BES with 0.5 V voltage, Bioc-0 was the biocathode BES with 0 V voltage.

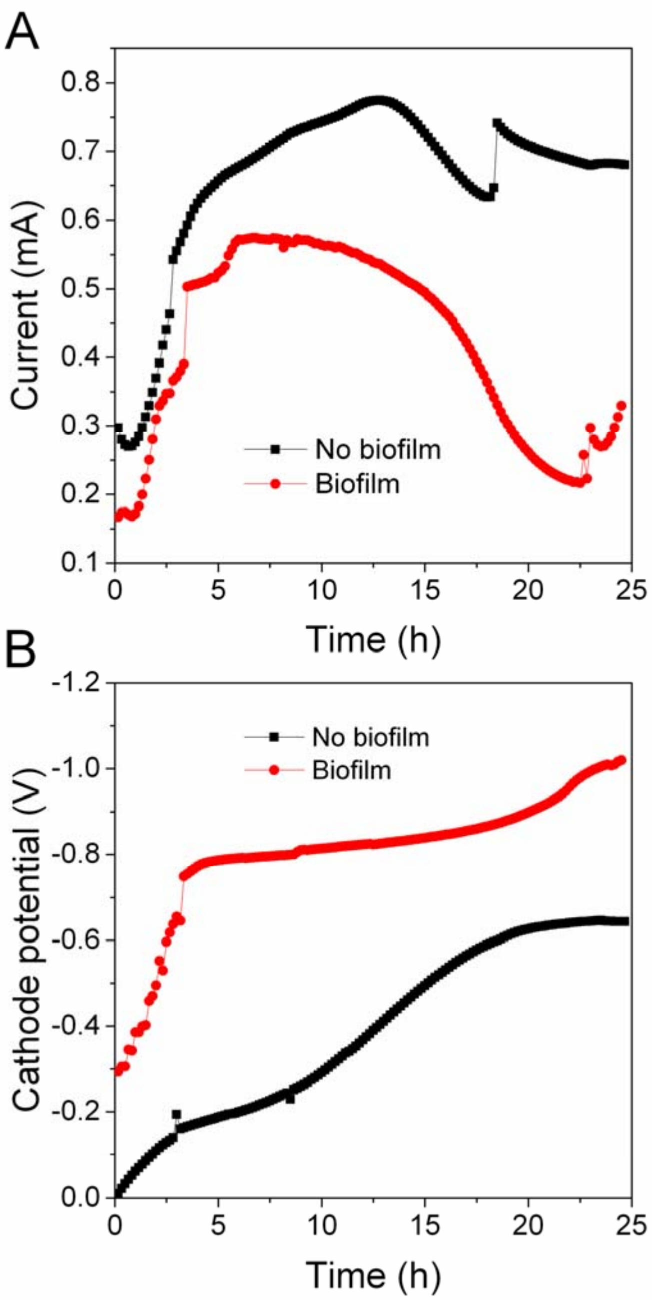

Figure S2: The current (A) and cathode potential (B) in biofilm and no biofilm of the cathode

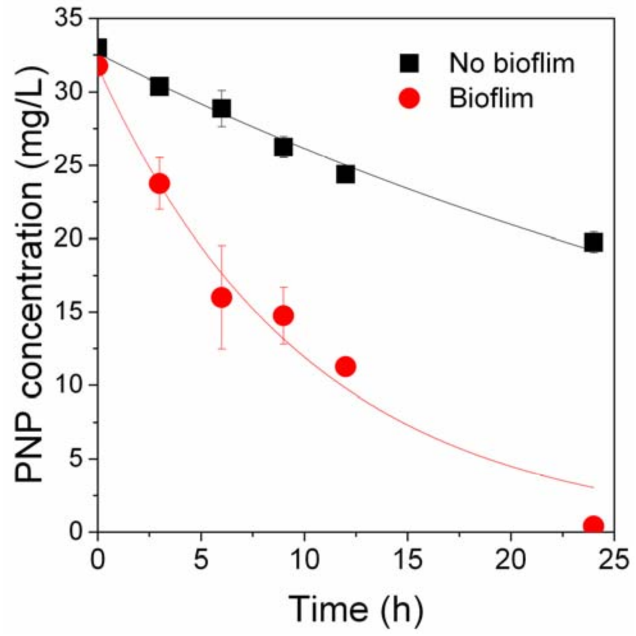

Figure S3: The PNP concentration in biofilm and no bioflim of the cathode

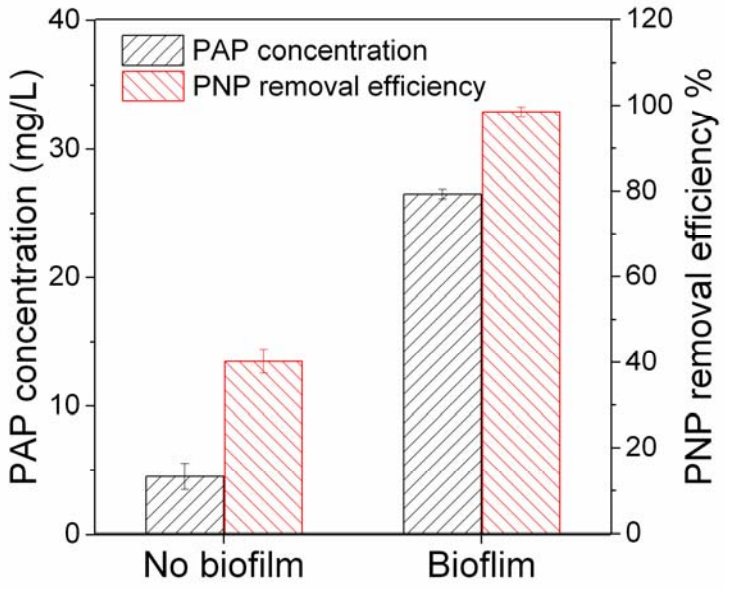

Figure S4: The PAP concentration and PNP removal efficiency at 24 h in biofilm and no biofilm of the cathode

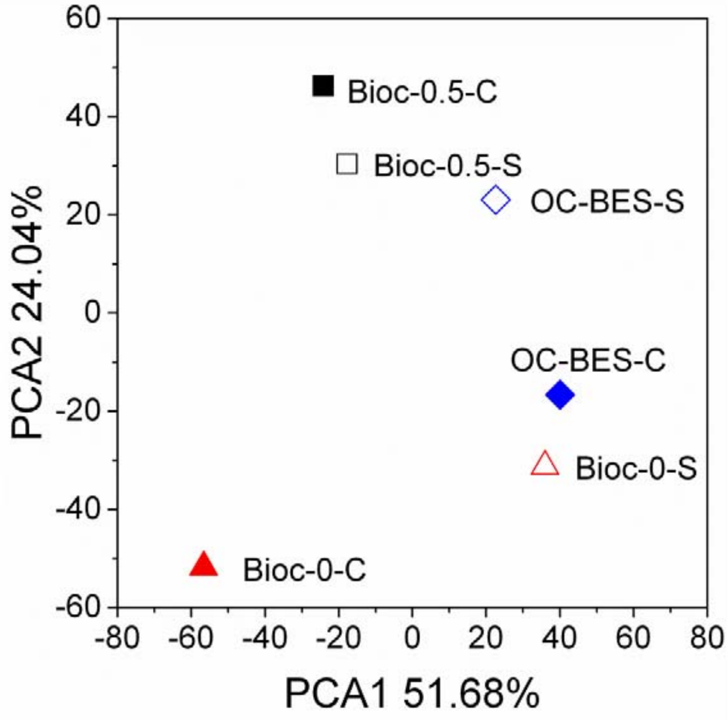

Figure S5: PCA of the cathode suspension and biofilm of biocahtode BES. The Bioc-0.5 was the biocathode BES with 0.5 V voltage, Bioc-0 was the biocathode BES with 0 V voltage, OC-BES was BES with open circuit. C represents cathode and S represents suspension.

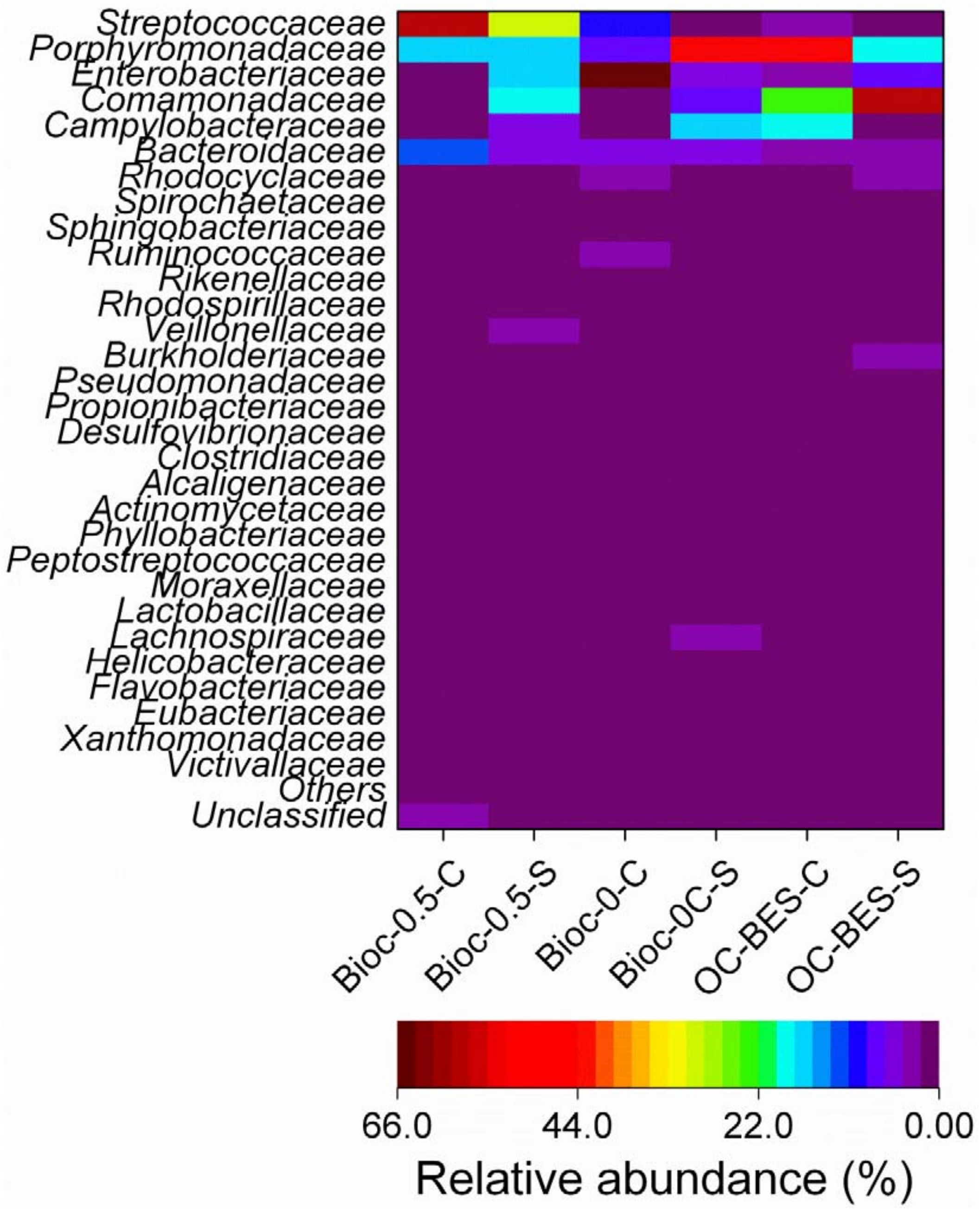

Figure S6: Heat map of the cathode suspension and biofilm of BES at family level. The “others” was the family less than 0.1% of the total summarized. The Bioc-0.5 was the biocathode BES with 0.5 V voltage, Bioc-0 was the biocathode BES with 0 V voltage, OC was BES with open circuit. C represents cathode and S represents suspension.

1.2 Supplementary Tables

Table S1: The respective k_{PNP}, R^2_{PNP}, $t_{1/2PNP}$, DE_{PNP}, k_{PAP}, R^2_{PAP} and FE_{PAP} of PNP degradation under different modes.

	k_{PNP} (h^{-1})	R^2 PNP	$t_{1/2}$ PNP	DE_{PNP} (%)	k_{PAP} (h^{-1})	R^2_{PAP}	FE_{PAP} (%)
Bioc-0.5	0.13±0.01	0.996	5.4	99.5±0.8	0.12±0.01	0.983	99.0±1.8
Bioc -0	0.024±0.002	0.977	28.4	60.9±0.05	0.051±0.012	0.967	62.4±4.5
OC-BES	0.013±0.0005	0.991	52.0	37.7±5.2	0.038±0.011	0.972	59.2±12.5

Table S2: Operation taxonomic units and community diversity indices of different mode biofilm based on 454 Pyrosequencing

	Reads	Shannon	Simpson	Chao	OTU	Coverage
Bioc-0.5-C	7833	3.04	0.178	800	440	0.972
Bioc-0.5-S	9337	3.61	0.0845	984	545	0.971
Bioc-0-C	10068	2.92	0.209	874	471	0.976
Bioc-0-S	10773	3.02	0.204	934	499	0.978
OC-BES-C	11312	2.82	0.19	909	504	0.977
OC-BES-S	11121	2.95	0.176	1019	528	0.975
